# A randomized trial of a minimal intervention for sexual concerns after cancer: a comparison of self-help and professionally delivered modalities

**DOI:** 10.1186/s12885-015-1638-6

**Published:** 2015-09-09

**Authors:** Janette Perz, Jane M Ussher, The Australian Cancer and Sexuality Study Team

**Affiliations:** Centre for Health Research - School of Medicine, Western Sydney University, Locked Bag 1797, Penrith, 2751 Australia

## Abstract

**Background:**

Information and discussion of sexual changes with a health professional is a high priority for many cancer patients in order to assist with sexual changes and ensure that sexual intimacy does not cease post-cancer. The PLISSIT model is widely recommended as a framework for providing sexual information and support, allowing for the discussion of sexual changes at various levels of increasing intensity. The aim of the present study is to evaluate the early stages of the PLISSIT model by examining the relative efficacy of written information provision about cancer related sexual changes, and information provision accompanied by a single session of counselling, for people with cancer and their partners, across a range of cancer types.

**Method:**

Eighty-eight people with cancer and 53 partners across a range of sexual and non-sexual cancers, took part in a randomised trial which adopted mixed method analysis to examine changes in psychological wellbeing, quality of life, relationship satisfaction and communication, and sexual functioning, following written information provision about cancer related sexual changes (self-help condition; SH), or written information accompanied by a single session of counselling (health professional condition; HP).

**Results:**

Ratings of the usefulness and efficacy of the SH and HP interventions, collected through analysis of Likert scales, open ended survey items and interviews, indicated that both conditions were found to be useful and efficacious by the majority of participants, serving to increase awareness of sexuality, improve couple communication about sex, and help in the management of sexual changes, through the exploration of non-coital sexual practices. In contrast, the quantitative analysis of standardized instruments found no significant improvements in psychological wellbeing, quality of life, relationship satisfaction and communication, or sexual functioning. There were significant reductions in self-silencing in the HP condition, and a trend towards increases in sexual satisfaction across both conditions.

**Conclusion:**

These results offer support for the early stages of the PLISSIT model, in terms of normalization and increased awareness of sexual changes after cancer, increased couple communication about sexual changes, and legitimation of exploration of a range of non-coital sexual practices and intimacy. However, more complex and intensive interventions are needed to address sexual functioning and psychological wellbeing. The findings provide support for the proposition that providing permission to discuss sexuality should be the core feature underpinning all stages of interventions designed to provide sexuality information and support for people with cancer and their partners, and also demonstrate the potential importance of limited information and specific suggestions.

**Trial registration:**

This study was registered in the Australian New Zealand Clinical Trials Registry.

(ACTRN12615000399594) on 29 April 2015.

## Background

There is a growing body of research examining the association between sexual changes experienced after cancer and quality of life or psychological wellbeing [1–5], with sexual difficulties associated with lower quality of life, and higher levels of distress. Information and discussion of sexual changes with a health professional is a high priority for many cancer patients [6, 7], assisting with sexual changes and ensuring that sexual intimacy does not cease post-cancer. This has led to the conclusion that health care professionals should routinely provide information about cancer related sexual changes, as well as the opportunity to discuss such changes in a holistic manner [8]. However, research evidence suggests that such discussions are not taking place with the majority of patients or partners [6, 7, 9, 10].

**Fig. 1:**
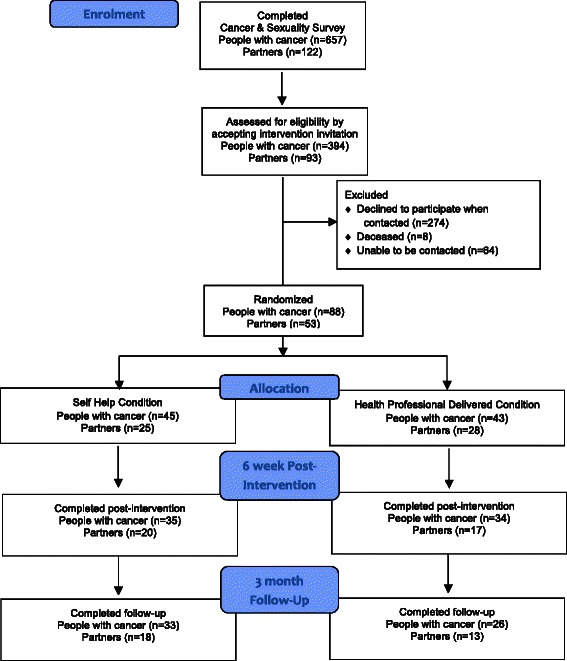
Participant flow diagram

In order to encourage patient-clinician communication about sexuality after cancer, a range of clinical practice guidelines [11–14] and sexual communication models [15–18] have been developed. The PLISSIT model [15], which advocates four levels of intervention - Permission, Limited Information, Specific Suggestions and Intensive Therapy - is widely recommended as a framework for providing sexuality information and support for people with cancer and their partners [17, 19–22]. This model allows health professionals to engage in the discussion of sexual changes at various levels of increasing intensity [23], starting with challenging the misconception that sexuality is ‘frivolous’ during cancer, by ‘giving permission’ for couples to talk about sex and be sexually intimate [24]. It potentially avoids providing information or interventions to people with cancer and partners that is not wanted or relevant [25], through encouraging the provision of ‘limited information’ in a written form, the preference of some patients [6]. If required, clinicians can provide ‘specific suggestions’ related to the adjustment to changes and expansion of sexual repertoires [26, 27], including sexual positioning or the use of sexual enhancement products [28, 29]. In contexts where there is a desire for ‘intensive sexual therapy’ or medical intervention, clinicians can refer to specialists for appropriate support.

Whilst there is a growing body of evidence demonstrating the efficacy of ‘intensive therapy’ to ameliorate sexual difficulties after cancer [for reviews see [30–32], to date, there has been no research systematically evaluating the early stages of the PLISSIT model, in particular, the impact of sexual information provision exclusively [30]. Written information about changes to sexuality after cancer has been used as a control condition [33], or as part of a broader intervention addressing sexual concerns [34, 35], however, in the latter case it is not clear what contribution information provision has made to patient or partner outcomes [30]. Equally, whilst sexual counseling has been a central component of a number of psycho-social interventions addressing sexual concerns after cancer [36–40], this has varied between three and six sessions, with no studies examining the efficacy of a single session offering ‘specific suggestions’ to address sexual concerns, as advocated in the early stages of the PLISSIT model.

Health professionals have been reported to be more likely to discuss sexual changes with individuals or couples experiencing a sexual cancer [10, 41]. With a few notable exceptions [e.g. [19, 42], previous research on the efficacy of interventions to address sexual concerns after cancer has also focused on cancers that directly affect the sexual or reproductive organs, such as prostate [31, 32], breast [30, 43] and gynaecological cancer [40, 44]. However, there is increasing evidence that both men and women across a range of cancer types report changes to their sexuality post-cancer [45], including lung [46], lymphatic [47, 48], colon [19, 49], non-Hodgkin lymphoma [50], head and neck [51], and colorectal cancer [42, 52]. This suggests that there is a need for research evaluating interventions to address sexual changes after cancer, across a range of cancer types. Previous reviewers of research evaluating interventions to address sexual concerns after cancer have also concluded that there is a need for “more methodologically strong research” in this field, as many studies do not use validated outcome measures, have a non-randomised design, and have a small sample size ([30], p.711).

In order to address these gaps in the research literature and evaluate the early stages of the PLISSIT model, the aim of the present study was to examine the relative efficacy of written information provision about cancer related sexual changes, and information provision accompanied by a single session of counselling, for people with cancer and their partners, across a range of cancer types.

## Method

### Design

The study used a randomised trial design and mixed method analysis to examine changes in psychological wellbeing, quality of life, relationship satisfaction and communication, and sexual functioning, following written information provision about cancer related sexual changes, or written information accompanied by a single session of counseling. Whilst randomised controlled trial designs have been described as the ‘gold standard’ [53] for evaluation of health interventions, there have been critiques of the sole reliance on such designs for evaluating health behaviour in a social context [54]. One way of addressing this critique is to include qualitative methods alongside standardised outcome measures [33, 34, 37, 44], allowing the impact of interventions to be quantified, at the same time as capturing subjective experience of taking part in the SH and HP interventions [55]. For this reason, a combination of quantitative and qualitative outcome measures was adopted in the present study, to assess participants pre-intervention, at six weeks post-intervention, and three months follow-up.

### Participants and procedure

Participants were eligible if they were over 18 years of age, and were a person with cancer, or the partner of a person with cancer who had experienced cancer related sexual concerns. Recruitment took place within a larger mixed methods study examining the construction and experience of changes to sexuality after cancer [45, 56, 57], with the study advertised nationally in Australia through cancer support groups, media stories in local press, advertisements in cancer and carer specific newsletters, hospital clinics, and local cancer organisation websites and telephone helplines. Six hundred and fifty seven people with cancer (535 women, 122 men) and 148 partners (87 women, 61 men) completed an on-line or postal questionnaire examining their experiences of sexuality and intimacy post-cancer (see Fig. 1). The sample consisted of a range of cancer types and stages, representing the major cancer types, but with a preponderance of breast and prostate cancer [see [45]. At the end of the survey, participants indicated whether they would like to be considered to take part in the evaluation of an intervention providing information about cancer related sexual changes. Those who agreed to take part were then randomly allocated to one of two conditions: self-help information only (SH), or information plus health professional consultation (HP). Randomization was stratified according to gender and cancer classification. The SH and HP interventions were offered on a couple basis for those in a relationship, following previous suggestions that couple based interventions are most effective [30]. However, in order to meet the needs of those who were single, a group over looked in previous research [31], or those who had partners who did not want to participate, the two interventions were also offered on an individual basis. All participants were explicitly encouraged to discuss the SH and HP intervention with sexual partners, and to share the written information with partners. Participants completed quantitative measures in a questionnaire pre-intervention at baseline, six weeks post-intervention and at three months follow-up. Ten participants from each condition, representing a cross section of gender, cancer type, and patient/partner status, took part in semi-structured interviews post-intervention.

Two individuals, a person with cancer and a partner, nominated by a cancer consumer organisation acted as consultants on the project, commenting on the design, method and interpretation of results. We received ethical approval from the University of Western Sydney Human Research Ethics Committee, and from three Health Authorities (Sydney West Area Health Service, South East Sydney Illawarra Health Service, and St Vincent’s Hospital, Sydney), from which participants were drawn. All participants were adults, and written informed consent was obtained in all cases.

### Quantitative measures

*Health related quality of life* was measured with the Medical Outcomes Study Health Survey Short Form (SF-12) [58] . This measure has been used to evaluate functional states in depressed, chronically ill and healthy populations. The SF-12 is comprised of 12 items, measuring two factors: mental health and physical health. Participants rate the degree to which their quality of life is compromised due to their health, on a series of Likert scales. High scores indicate a better quality of life. In this study, internal consistency calculated with Cronbach’s alpha = .71 and .74 for physical health for patients and partners respectively, and .72 and .75 for mental health for patients and partners, respectively.

*Psychological wellbeing* was measured with the Hospital Anxiety and Depression Scale (HADS) [59], a 14-item validated measure of anxiety and depression in non-psychiatric populations. Each sub-scale HADSA (anxiety) and HADSD (depression) has a maximum possible score of 21, with a score of between 8 and above recommended for “caseness”, the cut-off for clinical diagnosis. A score of 8–10 is categorised borderline and a score of 11 and above categorised as abnormal in relation to caseness [60]. Cronbach alpha coefficients for HADS-A = .83 and .79 for patients and partners, respectively; and for HADS-D = .82–80 for patients and partners, respectively.

*Self-silencing* was assessed with the Silencing the Self Scale (STSS) [61] a standardized scale consisting of 31 items measuring the extent to which individuals endorse self-silencing thoughts and actions in intimate relationships, using a 5-point Likert scale. In addition to a total score, the four subscales are: Care as Self-Sacrifice (e.g. Caring means putting the other person’s needs in front of my own), Silencing the Self (e.g. I don’t speak my feelings in an intimate relationship when I know they will cause disagreement), Externalised Self-Perception (e.g. I tend to judge myself by how I think other people see me) and The Divided Self (e.g. Often I look happy enough on the outside, but inwardly I feel angry and rebellious). High scores indicate greater self-silencing. In this study, the internal consistency of total STSS and subscales ranged from Cronbach alpha coefficients of .79–92 for patients and .65–87 for partners.

*Relationship satisfaction* was measured with the Brief Dyadic Adjustment Scale (DAS) [62], a 7-item validated instrument which examines relationship satisfaction and cohesion, using a 6-point Likert scale. Cronbach’s alpha = .88 and .75 for patients and partners, respectively.

*Sexual communication* was assessed with the Dyadic Sexual Communication Scale [63], a 13 item scale assessing perceptions of the communication process encompassing sexual relationships, using a 6-point Likert scale, with higher scores associated with better quality of perceived communication. Cronbach’s alpha coefficients = .91 and .89 for patients and partners, respectively.

*Sexual function* was measured with the Changes in Sexual Functioning Questionnaire (CSFQ-14) [64]. This is a 14 item validated instrument which provides a global measure of sexual functioning, using a 5-point Likert scale encompassing five domains identifying different aspects of sexual functioning: desire/frequency; desire/interest; arousal excitement; orgasm/completion; and pleasure. Scores are gender specific. Cronbach alpha coefficients for total CSFQ-14 scores of .87 for female patients and .89 for female partners, and .84 for male patients and .78 for male partners were found.

*Satisfaction with Sexual Relationship* was assessed with a one-item measure developed for the present study that asked participants ‘how satisfied are you with your sexual relationship?’, on a 5-point Likert scale.

### Qualitative measures

Participants were asked to respond to a series of open-ended questions about ways in which the SH or HP intervention: helped manage changes to sexuality; helped communication with partner about their sexual relationship; helpful and unhelpful aspects of the intervention; positive and negative consequences of the intervention. Ten participants in each condition also took part in a semi-structured interview, examining the experience of taking part in the intervention, and any perceived consequences in relation to sexual wellbeing. The interviews were audio-recorded ranged in duration from 20–45 min and were conducted on a telephone basis by a trained interviewer.

## Interventions

### Self-help booklet

The self-help information booklet was developed as part of the research project, based on accounts of sexual concerns experienced by people with cancer and their partners, consultation with health professionals, and examination of existing written resources on cancer related sexual concerns [65]. It consisted of 68 pages, which provided information about: what is sexuality and intimacy; how cancer and cancer treatment affects sexuality; the body and sex; sexuality across different stages of cancer; talking about sex and intimacy; information for partners and for single people; same-sex relationships; strategies for overcoming sexual concerns, including exploration of non-coital practices and intimacy; and support services available.

### Health professional intervention

The health professional consultation consisted of the self-help booklet followed up two weeks later by a one-hour telephone or Skype consultation between the participant and a counselor trained in discussing sexuality. Concerns or questions raised as a result of reading the booklet were discussed during the consultation, and suggestions for ameliorating sexual problems, based on the specific needs of participants, were offered.

## Analysis

### Statistical analysis

Univariate analyses were conducted to compare participants in the two intervention conditions for each of the socio-demographic variables of interest separately for people with cancer (PWC) and partners of people with cancer (PPWC). For continuous variables, one-way ANOVA were conducted with intervention, participant type, or gender used as the grouping variable, and the chi square test for independence used for frequency data. Descriptive frequency analyses were used to examine baseline/follow-up retention rates across intervention conditions and participate type. Descriptive analyses examined program evaluation measures. To allow for dichotomous analysis and facilitate interpretation, ratings of program usefulness were recoded into ‘not useful’ and ‘useful (a little > extremely)’, reflecting the direction and meaning of the original Likert scales. The Fisher’s Exact Test (FET) was performed upon the categorical data associated with the perceived usefulness of the booklet in managing sexual changes and communication about sexual changes across the SH and HP intervention conditions. In these analyses, the FET calculates the exact probability of significant differences in the reported assignments of participants in the two intervention conditions. Independent sample t-tests were performed on outcome variables to assess baseline differences between the SH and HP intervention conditions according to participant type. A series of separate mixed repeated measures ANOVA were conducted on scores on each outcome variable across the three time conditions, pre-test, post-test and follow-up as the within-subjects variables, with intervention condition (SH or HP), participant type (PWC or PPWC), gender (men or women) and cancer type (sexual or non-sexual) as the between-subjects factors. An alpha level of .05 was used for all statistical tests.

### Qualitative analysis

The interviews and open-ended questionnaire data were analysed using theoretical thematic analysis [66], using an inductive approach, with the development of themes being data driven, rather than based on pre-existing research on sexuality and cancer. In the analysis, our aim was to examine data at a latent level, examining the underlying ideas, constructions and discourses that shape or inform the semantic content of the data. All of the interviews were transcribed verbatim, and the answers to open ended questions collated. A research assistant read the resulting transcripts in conjunction with the audio recording, to check for errors in transcription. Detailed memo notes and potential analytical insights were also recorded during this process. A subset of the interviews and open ended questions was then independently read and reread by two of us (JU and JP) to identify first order codes such as communication about sexuality; legitimation of non-coital sex; liking information; intimacy more important than sexuality; normalising sexual change; increased awareness and reflection; acceptance of sexual change; importance of sex; validating sexual practice; no change; interventions not applicable; need for more specific information. There was a high level of agreement between coders, with any disagreement resolved by discussion. The entire data set was then coded by a research assistant using NVivo, a computer package that facilitates organization of coded qualitative data. All of the coded data was then read through independently by two of us (JU and JP). Codes were then grouped into higher order themes; a careful and recursive decision making process, which involved checking for emerging patterns, for variability and consistency, and making judgements about which codes were similar and dissimilar. The thematically coded data was then collated and reorganized through reading and rereading, allowing for a further refinement and review of themes [67], where a number of themes were collapsed into each other and a thematic map of the data was developed. In this final stage, a number of core themes were developed, which essentially linked many of the themes: increasing awareness and communication about sex; normalising sexual change; legitimation of intimacy and non-coital sex; lack of applicability and specificity. In the presentation of qualitative results, participant details are provided for longer quotes, including pseudonym, age, patient/partner status, type of cancer, and SH or HP condition.

## Results

### Participant profile

Eighty-eight people with cancer (47 women; 41 men) and fifty-three partners (28 women, 25 men) agreed to take part in the intervention arm of the study and completed the pre-intervention assessment, before being allocated into one of the two intervention conditions. Baseline participant demographic data is presented in Table 1 by self-help (SH) and health professional (HP) intervention groups for both people with cancer (PWC) and partners of people with cancer (PPWC). The sample was relatively equally gender split (53 % and 47 % respectively for women and men), predominately Anglo-Australian, and the average age of women (*M* = 52.7, *SD* = 11.5) was significantly younger than that for men (*M* = 59.8, *SD* = 11.4; *p* < .001). The majority of participants were heterosexual, partnered and living together, had an average length of current relationship of 24.2 years (*SD* = 16.3), and had participated in the study as a couple (73 %). Most participants had a sexual type (i.e. prostate, breast or gynaecological) cancer classification (81 %). Of the 141 participants who commenced the study, 92 completed both post and follow-up measures representing an overall retention rate of 65.2 %. The retention to follow-up was higher in the SH group (92.7 %) compared to 80.4 % for the HP group, but comparable for PWC (68.2 %) and PPWC (60.4 %).

**Table 1:** Baseline Sample Characteristics by Intervention Group for People with Cancer and Partners of People with Cancer

Characteristic	Self Help Package (SH) (*n* = 70) *M (SD)* or *n (%)*		Health Professional Delivered (HP) (*n* = 71) *M (SD)* or *n (%)*	
	Person with cancer (*n* = 45)	Partner (*n* = 25)	Person with cancer (*n* = 43)	Partner (*n* = 28)
Age:	56.9 (12.9)	55.7 (10.6)	55.6 (12.0)	55.4 (11.7)
Length of relationship (years):	22.1 (16.9)	27.8 (16.2)	23.6 (16.4)	25.5 (15.9)
Gender:				
Female	23 (51.1)	12 (48.0)	24 (55.8)	16 (57.1)
Male	22 (48.9)	13 (52.0)	19 (44.2)	12 (42.9)
Ethnicity:				
Aust/White European	43 (95.6)	23 (92.0)	40 (93.0)	25 (89.3)
Asian	2 (4.4 %)	1 (4.0)	-	2 (7.1)
Other	-	1 (4.0)	3 (7.0)	1 (3.6)
Relationship status:				
Partnered – Living together	30 (66.7)	21 (84.0)	32 (74.4)	26 (92.9)
Partnered – Not living together	6 (13.3)	4 (16.0)	5 (11.6)	2 (7.1)
Not in a relationship	8 (17.8)	-	6 (14.0)	-
Other/Not specified	1 (2.2)	-	-	-
Sexual identity:				
Heterosexual	45 (100)	24 (95.8)	41 (95.3)	-
Non Heterosexual	-	1 (4.2)	2 (4.7)	-
Cancer classification				
Sexual type cancer	35 (77.8)	20 (80.0)	36 (83.7)	23 (82.1)
Non-sexual type cancer	10 (22.2)	5 (20.0)	7 (16.3)	5 (17.9)
Intervention modality:				
Couple	26 (57.8)	24 (96.0)	26 (60.5)	27 (100)
Individual	19 (42.2)	1 (4.0)	17 (39.5)	-

### Program evaluation ratings

Evaluations of the booklet and health professional session elements of the program by intervention groups collected at follow-up are presented in Table 2. Participants in both groups rated the booklet favourably in terms of usefulness in helping to manage changes to sexuality and usefulness in helping with talking with their partner about changes to their sexuality. Participants in the HP group were significantly more likely to rate the booklet as being useful than those in the SH group. For those in the HP group, positive ratings of the session with the health professional were found with the majority reporting that the session was useful in both helping to manage changes to sexuality and in helping with talking to their partner about changes to your sexuality.

**Table 2:** Program Evaluations by Intervention Group

Measure	SH		HP		Test for group difference
	*n*	%	*n*	%	FET
Booklet usefulness in helping manage changes to sexuality					.013
Not useful	14	32.6	3	8.6	
Useful (a little > extremely)	29	67.4	32	91.4	
HP session usefulness in helping manage changes to sexuality					
Not useful	-		2	6.5	
Useful (a little > extremely)	-	-	29	93.5	
Booklet usefulness in helping communication with partner about the sexual relationship					
Not useful	19	44.2	6	20.0	
Useful (a little > extremely)	24	55.8	24	80.0	.045
HP session usefulness in helping communication with partner about the sexual relationship					
Not useful	-	-	3	12.0	
Useful (a little > extremely)	-	-	22	88.0	

### Group baseline comparisons

No significant baseline differences between participants in the SH and HP groups, or between PWC and PPWC were found for demographic measures reported in Table 1. No differences for PWCs in the SH and HP conditions in baseline outcome measures approached significance (*p* values ≥ .14). Similarly, no differences in outcome measures between the SH and HP conditions were found for PPWCs (*p* values ≥ .11).

### Changes in outcomes measures across time by intervention group and participant type

Separate three-way mixed design ANOVA were conducted with time (pre-test, post-test and follow-up) as the within-subjects factor, and intervention condition (SH or HP) paired with either participant type (PWC or PPWC), gender (male or female) or cancer type (sexual or non-sexual) as the between-subjects factors. No significant main effects or interactions with intervention condition were found for gender or cancer type. Baseline, post and follow-up means and *SD*s for outcomes measures in the SH and HP conditions for people with cancer are presented in Table 3 and for partners of people with cancer in Table 4. The main effects and interactions were not significant for physical health related quality of life, psychological wellbeing, sexual communication, male sexual function and satisfying sexual relationship, indicating that these measures did not significantly vary across the time conditions according to intervention condition or participant type. Significant within-subjects contrasts were found on the remaining measures as follows.

**Table 3:** Mean Scores for Outcome Variables Across Time Conditions by Intervention Group for People with Cancer

	People with Cancer					
Measure	Self Help Package (SH) (*n* = 33)			Health Professional Delivered (HP) (*n* = 26)		
	Pre *M (SD)*	Post *M (SD)*	FUP *M (SD)*	Pre *M (SD)*	Post *M (SD)*	FUP *M (SD)*
Health related quality of life						
Physical health	41.9 (11.1)	44.0 (10.1)	43.0 (13.5)	49.6 (10.6)	50.4 (7.2)	47.7 (9.9)
Mental health	50.0 (7.7)^#^	45.2 (12.0)^#^	45.8 (13.0)	51.2 (5.7)	49.3 (9.8)	50.0 (11.4)
Psychological wellbeing*						
Anxiety	10.9 (2.0)	10.4 (1.8)	10.9 (1.8)	10.9 (1.9)	11.0 (1.4)	11.2 (1.6)
Depression	9.1 (2.0)	8.9 (1.7)	8.7 (1.6)	8.6 (1.9)	8.6 (1.5)	8.7 (1.7)
Self Silencing*						
Care as self sacrifice	27.3 (7.0)	27.9 (6.0)	27.3 (5.9)	27.4 (7.3)	27.8 (7.7)	27.1 (8.2)
Silencing the self	25.4 (9.3)	25.8 (8.1)	25.1 (7.4)	26.7 (5.8)	25.3 (6.9)	25.9 (7.0)
Divided self	17.7 (7.3)	17.4 (6.2)	17.0 (6.5)	16.7 (6.1)	16.4 (5.8)	15.6 (5.5)
Externalized self perception	16.9 (5.9)	16.6 (5.9)	16.8 (5.4)	16.3 (5.2)^#^	15.4 (5.9)	14.9 (6.0)^#^
Total	85.7 (25.3)	86.3 (20.7)	85.3 (20.7)	86.9 (16.4)^#^	84.8 (19.6)	82.8 (20.3)^#^
Relationship satisfaction	31.0 (7.6)^#^	32.5 (6.8)^#^	33.1 (7.4)^#^	32.5 (7.3)	33.1 (6.6)	33.2 (4.0)
Sexual communication	46.7 (11.9)	45.4 (12.7)	46.8 (12.4)	49.1 (9.8)	49.7 (9.2)	51.4 (10.4)
Sexual function						
Female sexual function	38.2 (8.7)	38.2 (8.6)	37.5 (10.6)	35.2 (11.2)	33.2(12.5)	33.7 (12.4)
Male sexual function	33.4 (8.5)	35.1 (8.4)	33.8 (9.1)	33.9 (7.1)	34.4 (9.6)	34.5 (7.5)
Satisfying sexual relationship	2.5 (1.5)	2.8 (1.4)	2.8 (1.5)	2.6 (1.3)	3.0 (1.2)	3.0 (1.1)

^#^Significant difference between scores at *p* < .05; * Higher scores signify greater distress or greater negative impact

**Table 4:** Mean Scores for Outcome Variables Across Time Conditions by Intervention Group for Partners of People with Cancer

	Partner of Person with Cancer					
Measure	Self Help Package (SH) (*n* = 18)			Health Professional Delivered (HP) (*n* = 13)		
	Pre *M (SD)*	Post *M (SD)*	FUP *M (SD)*	Pre *M (SD)*	Post *M (SD)*	FUP *M (SD)*
Health related quality of life						
Physical health	51.8 (6.8)	52.50 (5.7)	52.0 (6.5)	50.1 (10.1)	49.8 (10.2)	47.1 (11.0)
Mental health	55.5 (4.5)	52.8 (8.5)	55.4 (6.2)	52.2 (5.7)^#^	54.1 (10.2)	57.1 (3.0)^#^
Psychological wellbeing*						
Anxiety	12.0 (1.5)	11.4 (1.3)	11.1 (1.6)	11.5 (1.9)	11.5 (1.8)	11.5 (2.1)
Depression	8.5 (0.9)^#^	8.9 (1.1)	9.1 (1.2)^#^	8.9 (1.4)	8.2 (1.1)	8.5 (1.2)
Self Silencing*						
Care as self sacrifice	26.4 (6.4)	27.9 (6.0)	27.3 (5.9)	27.9 (4.7)^#^	29.1 (5.5)	25.5 (5.8)^#^
Silencing the self	23.7 (7.9)	22.2 (6.7)	22.8 (8.2)	22.8 (6.3)	23.2 (7.0)	22.2 (7.4)
Divided self	12.6 (4.1)	13.2 (5.2)	11.8 (5.1)	14.4 (4.1)^#^	12.6 (3.0)^#^	12.2 (3.6)^#^
Externalized self perception	16.9 (5.9)	16.6 (5.9)	16.8 (5.4)	15.1 (4.5)^#^	14.1 (5.1)	12.1 (5.2)^#^
Total	78.0 (16.9)	77.4 (17.7)	76.9 (18.9)	80.2 (13.6)^#^	78.9 (14.4)	72.0(17.5^#^
Relationship satisfaction	36.8 (3.5)	36.2 (2.6)	36.2 (3.9)	35.8 (3.8)	36.2 (4.1)	37.1 (2.9)
Sexual communication	53.2 (10.1)	52.3 (9.6)	53.1 (10.6)	52.1 (8.8)	51.7 (7.5)	53.9 (8.9)
Sexual function						
Female sexual function	42.9 (8.8)	44.0 (5.9)	43.5 (4.4)	36.1 (10.1)^#^	32.0(10.7)^#^	35.1 (10.3)
Male sexual function	42.4 (8.5)	41.0 (8.7)	41.6 (7.8)	36.7 (5.0)	38.7 (9.0)	34.0 (9.5)
Satisfying sexual relationship	3.1 (1.3)	3.3 (1.2)	3.5 (1.2)	3.1 (1.3)	3.5 (0.9)	3.5 (1.0)

^#^Significant difference between scores at *p* < .05; * Higher scores signify greater distress or greater negative impact

For people with cancer in the SH condition, improvements over time were found for relationship satisfaction (*F* = 9.61; *p* = .004; *n*^*2*^*p* = .26 [level 2 vs. level 1]), but decreases in mental health related quality of life (*F* = 5.27; *p* = .03; *n*^*2*^*p* = .16 [level 2 vs. level 1]) were also reported. PWCs in the HP condition reported decreases over time in scores for total self-silencing (*F* = 5.14; *p* = .03; *n*^*2*^*p* = .05 [level 3 vs. level 1]) and externalized self-perception (*F* = 7.91; *p* = .009; *n*^*2*^*p* = .06 [level 3 vs. level 1]), indicating reduced self-silencing.

For partners of people with cancer in the SH condition, depression scores significantly increased from baseline to follow-up (*F* = 5.12; *p* = .04; *n*^*2*^*p* = .23 [level 3 vs. level 1]). In the HP condition, female sexual functioning scores decreased significantly from baseline to post (*F* = 7.84; *p* = .02; *n*^*2*^*p* = .47 [level 2 vs. level 1]), but returned to baseline levels by follow-up. For PPWCs in the HP condition, decreases in scores over time were found for total self-silencing (*F* = 8.46; *p* = .01; *n*^*2*^*p* = .41 [level 3 vs. level 1]), care as self-sacrifice (*F* = 10.43; *p* = .007; *n*^*2*^*p* = .47 [level 3 vs. level 1]), divided self (*F* = 6.75; *p* = .02; *n*^*2*^*p* = .36 [level 2 vs. level 1]) and (*F* = 6.80; *p* = .02; *n*^*2*^*p* = .36 [level 3 vs. level 1]), and externalized self-perception (*F* = 6.88; *p* = .02; *n*^*2*^*p* = .36 [level 3 vs. level 1]).

## Qualitative results

### Increasing awareness and communication about sex

Sexuality and intimacy are often positioned as trivial activities in the context of cancer, which can leave couples reluctant to raise sexual concerns with each other, or with health professionals [9, 68]. The majority of participants, both partners and people with cancer, reported that taking part in the SH or HP intervention increased their awareness of sexual issues as evidenced by the following accounts: “improved awareness of both our sexual needs”; “gave starting point for discussion”; “made me think about it more”; opened up new areas to discuss – also to understand”; “made me aware of possible problems”; brought sex into focus and put it on the agenda”. This awareness was reported to have served to challenge “taboos” and legitimate sexuality as an issue of concern following cancer diagnosis and treatment, in a context where participants reported that “sexual dysfunction was never mentioned as a side effect of cancer diagnosis”, and they “were never informed about possible negative side effects such as not being able to experience penetration”. Awareness also served to facilitate communication about sexual matters within relationships, as Simon (age 53, patient, sarcoma, SH) commented:

I think, in that people find sexuality a difficult thing to talk about, or an embarrassing thing to talk about, or it’s very low on your list of priorities when you are undergoing chronic illness. But, there’s a reminder there that it shouldn’t be that sort of threatening, that it an important part of an ongoing, loving relationship you have with somebody, would be it your wife or partner or lover or whatever it is.

Increased communication and “being more open with each other about sex” was predominately reported in long-term relationships. For example, Ewan (age 64, prostate, HP), described having had a “normal and good sex life” in his 45 years of marriage, but that the HP intervention: “helped us to talk a little more about sexual things”; and Jason (age 35, partner, non-Hodgkin’s lymphoma, HP) said that “there were still a few things that (he) hadn’t yet got around to asking” his partner of 12 years, but that the HP intervention “actually saved me from having to ask, or when I did ask, um, it was more around, ‘so is this what happened with you?’”. However, a number of participants also reported increased communication in the context of a new relationship, as evidenced by Zoe’s account (age 48, patient, breast, HP):

I just had just gone into a new relationship about eight months ago and it just gave me something to – and this person wasn’t with me when I was going to my treatment, so it was just a really good springboard for me to open up discussion with him about where I’m at sexually.

There were also a number of accounts of anticipating using the information booklet in future relationships, as a means of facilitating discussion of sexual issues, as is evident in Boris’s account: “you could make your partner aware and just say, look, you know, there’s something for you to read if you want a relationship with me. These are my issues. Or some of my issues” (age 61, patient, prostate, SH). The SH and HP interventions were also reported to have facilitated discussion with health professionals involved in the ongoing care of patients, including requests for ‘assistive aids’ [69]: “helped with discussing sex with my oncologist”; “I went to my GP for creams and soreness which helped a bit”; “helped me to ask for penile injections”; “dealt with my erectile dysfunction which finally led to my having a prosthesis”.

Whilst there were many accounts of the information booklet acting as a “starting point for discussion”, or “getting us to talk”, participants who took part in the HP condition described the additional health professional consultation as further facilitating couple discussion of concerns: “The booklet opened up ways to talk about my issues with my partner, the f/up consult gave the courage to do so” (Helen, 56, patient, breast, HP); “the book got us to discuss things that we probably hadn’t discussed previously…the meeting did help us to iron out a few little problems that we may have had” (Will, age 73, patient, prostate, HP); “provided an opportunity to hear the partner’s opinion from a third party’s questions” (Hal, age 63, patient, prostate, HP); “because we were able to discuss sex with a third person, made it easy to talk more openly with each other (David, age 72, patient, prostate, HP). The professional consultation was also described as having made sections of the booklet “a lot more relevant”, and allowing participants to “take some concepts from the book and then talk about how it is exactly for me”, with subsequent couple conversations serving to “clarify issues”.

### Normalizing sexual change

Absence of either discussion or information provision regarding cancer related sexual changes can lead to feelings of isolation, or the belief that sexual difficulties are unique to the individual [6]. Many participants reported that the HP or SH intervention served to normalize their experience of sexual difficulties, “reassuring” participants that sexual changes were “OK and normal” or a “common problem” and that they are “not the only one this is happening to”; as “others feel the same way and there are solutions”. This sense of “normalcy” undermined isolation experienced by some participants, as is evident in the following accounts: “I don’t feel so alone”; “I’m not the only one”; “it was useful to read about what other people have gone through”;

I found the recognition that there are problems with sexuality with cancer very helpful. It is better to be in a boat with others rather than trying to paddle alone through rough waters (Emma, age 68, patient, breast, HP).

Normalizing sexual changes was also reported to reduce concerns, leading to “not feeling so guilty about lack of sex”, or “less guilty about not wanting sex”, as well as giving “a greater sense of not worrying about it”, and confirming that “there is nothing shameful and embarrassing about the condition”. In this vein, many participants reported that they wished they had “had this information at the beginning of treatment”, and it was important for such information to be provided for others “from the get go” in order minimise such concerns:

The thought process doesn’t get around to, “Could this be part of my illness,” until much later on, whereas if they’re given that booklet from the get go it could be quite beneficial because then they can sit down and go, “Okay, then this is part of it too.” And it may well save couples from the angst and anguish of, “Is it me or is it him, is it her, is it – is it?” You know, “Because of the treatment, is it something I’ve had for a while but we haven’t discussed, ‘what’s the problem?’ (Jason, age 35, partner, non-Hodgkin’s lymphoma, HP).

This process of normalization was also reported to lead to greater acceptance of sexual and embodied change after cancer, as well as a greater sense of comfort in the limitations of the body: “it allowed me to accept some of the changes to my body and its function”; “I was really starting to wonder if it’s just me, but I now am far more comfortable and I think my partner is, this is how I am and that’s ok” (Zoe, age 48, patient, breast, HP).

### Legitimation of intimacy and non-coital sex

The ability or desire to engage in coital sex is often compromised following cancer treatment, as a result of men’s erectile dysfunction [70], diminished genital size and urinary incontinence [71, 72], loss of sexual desire [73], decreased orgasmic sensation, and bowel and urinary incontinence [74]; or women’s pelvic nerve damage, clitoris removal, vaginal stenosis, and fistula formation [75], fatigue [76], dyspareunia [77], or vaginal dryness [78]. Many couples cease engagement in any sexual activity if coital sex is not possible [9], as sexual intercourse is conceptualised as ‘real sex’, a phenomenon described as the ‘coital imperative’ [79]. However, the ability to develop ‘flexible’ sexual practices [80, 81], or to ‘renegotiate’ sexual behaviour to include non-coital sex and non-genital intimacy [57], can allow couples to maintain sexual activity and intimacy after cancer. Many participants reported that taking part in the SH or HP intervention acted to encourage the development of non-coital sexual activity and intimacy, with positive consequences for their sexual and intimate relationship. For example, Grace (age 65, partner, prostate, SH) said that the discussion of “intimacy (as) different to sexuality” in the booklet “was very good, and encouraged the touching and listening”. Others talked of “being reminded that you can have intimacy that doesn’t involve sex”; “my husband seemed somewhat relieved to just kiss and cuddle”; “the act of intimacy has found a place in our relationship”; “the appreciation of just touching each other was good”. A number of participants also gave accounts of having their non-coital sexual practices validated through the HP intervention, confirming that it was “the right thing to do”.

Sex has not been easy since I had the prostatectomy. Hasn’t been easy, and erections are not easily come by. And the kind of intimate things we’ve been doing with each other, you know, playing, and you know manipulating, it – we didn’t know whether that was quite the right thing to be doing. But talking with HP and reading the book, we clearly believe now that we haven’t been doing anything that we shouldn’t be doing (Will, age 73, patient, prostate, HP).

This account reflects potential self-judgement relating to the “right” kind of sex, which might be more common in contexts where individuals were taught that masturbation was “wrong”. This is made explicit in Simon’s account, below:

I liked the openness of, the whole point of caressing one another as a – as an adjunct to making love. Masturbation – it was good – handled well, I thought. Maybe that’s because I’m coming from a culture where you are told that was wrong, you know? And that’s - to get people to think that that’s okay, that exploring and knowing their bodies and knowing and helping your partner to, um, know what is enjoyable, was really good to keep in and to, um, emphasise (Simon, age 53, patient, sarcoma).

Imogen (age 74, partner, sarcoma, SH) also reflected this position in her discussion of herself as “old-fashioned” and “naïve” in relation to sexuality, reporting that the SH intervention “opened my eyes to a lot I didn’t know”, which she said was very helpful. Other participants talked of having a “veil lifted” in relation to sexuality; feeling more confident in exploring sex toys, “now knowing it’s OK if I do this”; or feeling legitimated in entering a sex shop: “next time we pass one or go near a sex shop we would most likely enter without feeling shy” (John, age 72, patient, prostate HP). As a result of this legitimation of non-coital sex, which led to renegotiated sexual practices, a number of participants described finding that “the sexual and physical intimacy is much better”, which “has been a very positive experience, and realising that “intimacy is more important than orgasm and ejaculation”, or that “even though full erection is not always possible, mutual stimulation still gives us both full satisfaction”.

### Lack of applicability and specificity

Whilst all of the interviewees were positive about the SH or HP intervention, there were a number of negative comments in the open-ended questionnaire responses, suggesting that the information contained in the booklet was not applicable to all participant concerns. In a number of cases, lack of applicability was described as being because the SH or HP intervention could not alter the “medical outcome” of embodied sexual changes, such as erectile problems or vaginal dryness: “I feel it is hard to try to fix the erectile dysfunction”; “my only problem is gaining normal erectile functioning after a radical prostatectomy… the intervention is unlikely to affect my medical outcome”; “no change in vaginal situation”; “it didn’t help as my chemo is not helping and I am very dry”. Others described entrenched or “extreme” sexual problems which could not be addressed: “information not for extremes like mine”; “I have always had issues sexually. Cancer has just added onto those issues”; “nothing is better so we just accept it”. Lack of attention to specific difficulties was also identified by some: “the booklet was not directed at my problem at all”; “not really specific enough for someone with bladder cancer”; “there was not enough about male sexual problems”; “I am pleased that same-sex men were catered for- but some of the sections on sex were too vague or not useful”. Two participants described the information contained in the booklet as “embarrassing”. The inability of written information to address the magnitude of distress experienced as a result of sexual changes was also commented upon:

I feel quite devastated by the cancer when it comes to my sex life. I don’t think a chirpy booklet can address the sense of loss that I have experienced (Cara, age 47, patient, breast).

These accounts provide some insight into rationale behind the rating of the SH or HP interventions as ‘not useful’ by a minority of participants, on the Likert scales.

## Discussion

The PLISSIT model [15] has been widely adopted to address the need for information and therapeutic intervention to address sexual concerns experienced after cancer. The purpose of this study was to evaluate the relative efficacy of minimal interventions recommended at the early stages of this progressive model: ‘permission’ and ‘limited information’ provided through a written booklet, compared with ‘specific suggestions’ provided in a counselling session, in addition to the written information. Our adoption of a mixed method approach allowed us to evaluate significant changes pre-post intervention and at follow-up across conditions, as well as the subjective experience of why each intervention may be helpful or unhelpful, providing insight into the reasons why such interventions might work.

Ratings of the usefulness and subjective efficacy of the HP and SH interventions, collected through analysis of Likert scales, open ended survey items and interviews, indicates that both interventions were found to be useful and efficacious by the majority of participants, serving to increase awareness of sexuality, improve couple communication about sex, and help in the management of sexual changes. This suggests that both written and health professional delivered interventions meet the aims of the early stages of the PLISSIT model, in providing permission, limited information and specific suggestions to address sexual changes after cancer. The addition of the consultation with the health professional produced higher ratings of usefulness, which was reflected in the qualitative accounts of the benefit of the consultation. This suggests that a minimal intervention, in terms of one-off session of counselling, may provide greater benefit than written information alone in addressing sexual changes after cancer for many individuals. However, previous research has reported that whilst some individuals with cancer prefer consultation sessions about sexual changes [82, 83], others prefer written information [6], or a combination of the two [84, 85], with the need for consultations being higher close to diagnosis, and preference for written information increasing over time [86]. The use of a randomised trial design in the present study did not take these preferences into account, which may explain some of the attrition at follow-up, as the mode of intervention may not have been the preference of the participant. Future research in this field could address this issue through utilizing a patient preference design, which has the benefit of closely mirroring everyday clinical practice, overcoming the limitation of applicability to clinical practice in an RCT design [54].

In contrast to positive accounts of the interventions presented in the qualitative data, the quantitative analysis of standardized instruments did not find significant improvements in depression and anxiety, quality of life, and couple sexual communication, following the interventions. These findings confirm previous reports of an absence of an effect of psychosocial interventions aimed at addressing post-cancer sexual changes on quality of life and psychological wellbeing [39, 87]. People with cancer in the SH condition reported a significant improvement in relationship satisfaction; however, this was not reported by patients in the HP condition or partners, reflecting the findings of previous research on the impact of interventions [27, 36, 88]. Psychological wellbeing, quality of life and relationship functioning, for both patients and their partners, is influenced by many factors, across physical, psychological and social domains [89–92]. Written information or minimal psychosocial interventions might thus be expected to have little impact upon wellbeing and relationships, unless such interventions are part of a broader program of psychosocial support.

Sexual functioning is the primary outcome measure used in psychosocial interventions to address sexual concerns after cancer [30–32]. In the present study, we found no significant improvement in sexual functioning following the intervention, as has been reported in previous research evaluating psychosocial sexual interventions post-cancer [34, 93, 94]. In contrast, a number of studies have reported a positive impact of psychosocial interventions on sexual functioning after cancer, for women partners of men with prostate cancer [36], men with prostate cancer [31], women with gynaecological cancer [40, 95] or breast cancer [30], and couples affected by colorectal cancer [42] . There is also some evidence of a positive impact of such interventions on psychological wellbeing [44] . In the main, the interventions which had a positive impact in these domains were more intensive than those evaluated in the present study, offering a number of sessions of one-to-one intervention, drawing on principles of sex therapy and psychological therapy, leading to the conclusion that it is more “complex designs” that have a “positive effect” [31], p.2012] on sexual functioning.

An improvement in sexual satisfaction across both intervention conditions, for both people with cancer and partners, which was maintained at follow-up, was observed, supporting previous research reports [19, 33, 36, 44], although levels marginally failed to reach statistical significance. This apparent contradiction between sexual functioning and satisfaction ratings may be explained through qualitative accounts of sex having being legitimated as a concern, and successful renegotiation of sexual practices to include non-coital intimacy, following engagement with the intervention. In the present study, this might have resulted from the specific focus on the development of intimacy and non-coital sexual practices in contexts where coital sex was difficult or no longer possible. A previous study evaluating a four session telephone intervention with a similar focus on the development of intimacy after colorectal cancer [42] reported a positive effect on self-efficacy for enjoying intimacy. This theme was evident in qualitative accounts in the present study, where participants reported feeling more confident about talking about sex with their partner, as well as exploring ‘flexible’ [80] sexual practices, such as touching, kissing, and sex toys.

In combination, these findings suggest that the primary aim of information and psychosocial interventions to address sexual changes after cancer should not be to return individuals to the level of sexual functioning that was enjoyed before cancer, as this is difficult for many to achieve, particularly when clinical levels of sexual difficulties are reported, as was the case in the present study. A focus on improving intimacy, sexual flexibility, and renegotiation of sex is a more achievable outcome for many individuals experiencing embodied sexual changes after cancer [57, 80]. These renegotiated sexual practices can be experienced as positive and enjoyable, and in some instances as an improvement in sexual intimacy and pleasure [57], as evidenced by the qualitative accounts of some of the participants in the present study. This suggests that non-coital sexual expression and intimacy should not be positioned as a poor substitute, or inferior, to ‘real sex’, as is often the case in broader cultural discourse about sexuality [96, 97]. Participants who adopted this ‘coital imperative’ discourse [79], p232] in the present study were less likely to explore non-coital intimacy or sexual practices, and more likely to report that the intervention was not useful for them. For those who could effectively utilise assistive aids, such as penile implants or injections, this was not problematic; however, assistive aids are not effective for many individuals [57, 69], and the absence of exploration of non-coital sexual practices can lead to sexual abstinence, with negative consequences for well-being and couple relationships [9].

Other than higher ratings of usefulness in the HP condition, the only significant difference found between the two levels of intervention in the quantitative analysis was the significant reduction in self-silencing reported by patients and partners in the HP condition. Self-silencing is characterized as the propensity to engage in compulsive caretaking, pleasing the other, and inhibition of self-expression in relationships, in an attempt to achieve intimacy and meet relational needs [98]. However, this can lead to a self-division between an “outwardly conforming and compliant self” and an “inner self who is angry and resentful” [99], p.177]. Findings of a reduction in self-silencing following the HP intervention suggests that patients and partners are less likely to feel externally judged about sexual issues, and more likely to express their own sexual needs. The absence of communication about sexual changes after cancer has been found to a significant predictor of sexual dysfunction [45]. In contrast, couples experiencing cancer who are mutually responsive, attend to each other’s needs, and talk openly about their difficulties, have been found to be more able to engage in effective emotion and problem focused coping [100], which allows them to find benefits in the cancer experience, such as personal growth and relationship closeness [101, 102]. This pattern of mutual communication has also been found to be associated with lower levels of distress for patients and partners, and higher levels of marital satisfaction [103–105]. The findings of the present study suggest that minimal interventions that involve a consultation component may be more effective in encouraging communication about partner sexual needs and concerns, as well as reducing partner self-silencing, with potential benefits for both the sexual relationship, and the development of emotional intimacy within the couple.

There have been suggestions that couple based psychosocial interventions that contain an element of sexual therapy are the most effective modality for addressing sexual changes after cancer [30]. However, the present study did not find a consistent or significant difference between individual and couple administration of the interventions, which supports the findings of two previous studies that systematically compared couple and individual psychosocial sexual interventions in the context of cancer [36, 106]. This suggests that the early stages of the PLISSIT model can be effective if offered on either a couple or individual basis. Our qualitative finding that the interventions had a positive impact on couple sexual communication and sexual exploration suggests that partners were engaging with the intervention, regardless of whether they were formally part of the study. Combined with previous reports that that psychosocial interventions are more effective when partners engage with the process through taking part in homework activities [36, 107], this suggests that clinicians should include an emphasis on couple communication and exploration of sexual changes, regardless of whether the intervention is individual or couple based.

The strengths of this study were the use of a randomised trial methodology, and a combination of standardized quantitative and qualitative measures, evaluating participants at pre-intervention, post-intervention and follow-up. The inclusion of partners as well as people with cancer, across a range of cancer types, is also a strength of the study, further demonstrating that sexual changes affect individuals with both sexual and non-sexual cancers, and that minimal interventions can be perceived to be effective in both contexts. One of the limitations of the study was the high level of sexual dysfunction in the sample with all participant groups except women partners in the SH condition scoring below sexual functioning thresholds. This may have rendered the minimal interventions recommended at the early stages of the PLISSIT model ineffective, particularly in relation to sexual dysfunction. Further research evaluating the early stages of PLISSIT is needed to with individuals with sexual concerns that do not meet clinical criteria. A second limitation of the study was the absence of a control group who did not receive any intervention. Whilst this decision was made for ethical reasons, a future study could include a wait list control group, who receive the intervention after the assessment is complete. The over representation of sexual cancers, primarily breast and prostate cancer, and under-representation other common non-sexual cancer types, including respiratory, skin, gastro-intestinal and head and neck cancers, is also limitation. This may be because individuals with non-sexual cancers are less likely to volunteer for a study on sexual changes. Further research is needed to examine interventions to address sexual concerns in individuals with non-sexual cancers.

## Conclusions

Despite limitations, this study offers support for the early stages of the PLISSIT model, in terms of normalization and increased awareness of sexual changes after cancer, increased couple communication about sexual changes, legitimation of exploration of a range of non-coital sexual practices and intimacy, and a trend towards increased sexual satisfaction. Whilst more intensive therapy may have been effective in addressing sexual dysfunction, psychological wellbeing, and relationship satisfaction, as suggested in the later stages of the PLISSIT model, it has previously been suggested that the first stage of PLISSIT, providing permission to discuss sexuality, should be the core feature underpinning all stages of intervention [108]. The findings of the present study provide support for this viewpoint, and also demonstrate the potential importance of limited information and specific suggestions from the perspective of people with cancer and their partners, across sexual and non-sexual cancers.

## Abbreviations

ANOVA: Analysis of variance
HP: Health professional condition
SH: Self-help condition
PWC: Person with cancer
PPWC: Partner of a person with cancer
